# p53 and aging: role of p66Shc

**DOI:** 10.18632/aging.100583

**Published:** 2013-07-18

**Authors:** Enrica Migliaccio, Marco Giorgio, Pier Giuseppe Pelicci

**Affiliations:** ^1^ European Institute of Oncology, Via Ripamonti 435, 20141-Milan, Italy; ^2^ Dipartimento di Medicina, Chirurgia e Odontoiatria, University of Milan, 20142-Milan, Italy

The p53 protein was discovered many years ago as a tumour suppressor gene and despite the wealth of information that has accumulated, a complete understanding of how p53 functions remains still elusive.

p53 is a key regulator of the checkpoint response to DNA damage in mammal cells and is affected by loss-of-function mutations in the majority of human cancer. In various normal adult tissues, basal levels of p53 protein are low, but specific stress signals induce post-translation modifications and its stabilization, leading to activation of distinct transcriptional programmers. In response to stress signals p53 mainly acts to repair DNA damage and depending on the severity of the damage, the cell microenvironment and the cell type, p53 can orchestrate different cellular outcomes such as apoptosis and senescence.

In addition to its nuclear activity, p53 also possesses biological activities that are transcription-independent, indeed p53 functions also in the mitochondria and cytoplasm, where, depending on the kind of stress either triggers apoptosis or inhibits autophagy [1, 2].

Then, the DeltaNp53 isoform, that lacks the transactivation domain, contributes to regulate p53 functions [3]. Notably, the over-expression of DeltaNp53 (p44) in mice affects IGF-1 induced gene expression and accelerates aging [4]. Cells from these mice are more susceptible to both p53-mediated apoptosis and senescence, suggesting that enhanced tumor suppressive response comes at the cost of accelerated aging.

**Figure 1:**
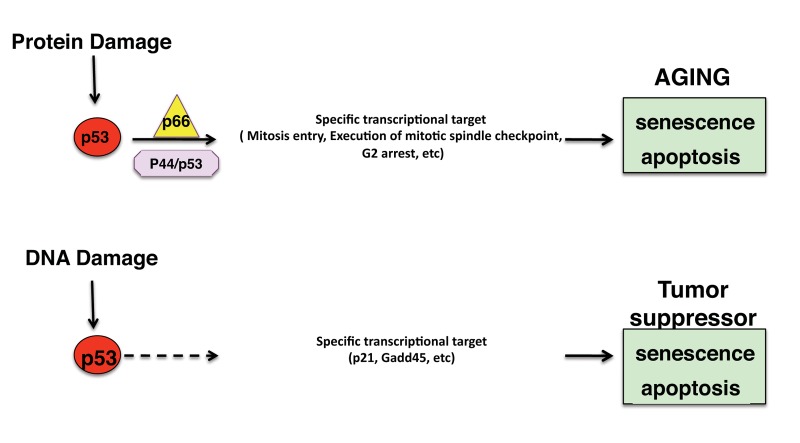
Distinct p53 pathways regulate tumour suppression and aging. Oxidative stress activates a specific specific transcriptional response, mediated by p66Shc and p44/p53 isoforms, which regulates cellular senescence and aging.

Indeed, emerging evidence suggested that p53 and DNA damage checkpoints have also a prominent but still not well characterized role in the regulation of aging in worm, mice and human [5]. How p53 differentiate between different stresses so that its activation leads to the correct response and whether aging is part of the tumorsuppressive function of p53 are outstanding questions. Emerging findings suggest that p53 activity on specific target is regulated by specific p53 isoforms, in the context of specific activating-signals [3]. Typically, p53-dependent growth arrest in G1 phases of the cell cycle have been attributed to transcriptional activity of p53 [both activation and repression] but contrary to the p53-transcription activation the biological significance of p53-mediate transcription repression and its role in G2/M arrest of the cell cycle is still largely undisclosed. p44/p53 (p47 in human) is an N-terminally truncated p53 isoform, which forms homo-oligomers and hetero-oligomers with p53, and induces G2/M cell-cycle arrest in response to serum deprivation or endoplasmic reticulum (ER) stress [6]. However, the regulation and physiological role of this p44 isoform are just starting to become known and its pro-aging function remains the unique indication regarding its putative physiological role [4]. Recent studies have demonstrated the presence of internal ribosome entry site (IRES) sequences in p53 mRNA that mediated the translation of both full-length and p44/p53 isoforms and represent a novel control of p53 gene expression and activity [6]. Then, the p44 isoform translation was found boosted by ER stress and plays an essential role in the unfolded protein response (UPR) that leads to a p53-mediated G2 cell cycle arrest [6]. Recently, it has been demonstrated that p53-dependent G2 arrest following ER stress is mediated by the p44 isoform [6]. One intriguing open question on p53 role in life span determination comes from the assumption that the increase of p53 activity is associated at acceleration of aging and at decrease of cancer incidence, and in opposite way, the attenuation of its activity correlates with short life span and increase of incidence of cancer. However, contrary to this idea, it appeared that many of the long-lived strains models, including p66Shc knockout mice [7], attenuation of p53 function does not correlated with increased of cancer. Recent findings suggest also that p53 and p66Shc are genetically and functionally linked [8]. p66Shc is a vertebrate protein whose targeted deletion in mice induces resistance to a variety of aging-associated diseases, including obesity, atherosclerosis, ischemic injury and diabetes, and extends lifespan [7]. In mitochondria, p66Shc uses reducing equivalents of the electron-transfer chain via direct oxidation of cytochrome c to generate pro-apoptotic reactive oxygen species [ROS], acting as a stress-induced red-ox enzyme, that also function as signaling molecules to activate specific targets [7]. Our group demonstrates that the p53 transcriptional-response to oxidative stress involves a large number of G2/M-mitosis genes and depends on p66Shc. In particular, the expression of p66Shc is indispensable for the function of the p53 isoform (p44/p53) to induce of G2/M cell cycle arrest, after oxidative stress. Consistently, p66Shc deletion decreased some of the signs of premature aging observed in mice overexpressing the p44 isoform [8], such as, decrease lifespan, decrease of fertility, premature accumulation of fat tissue in the liver, premature thymic involution and alopecia, demonstrating that p66Shc is critical for the biological effects of p44/p53 also in vivo [8]. Based on these results we hypothesized the tumour suppressive and the pro-aging functions of p53 are managed by two distinct pathways (Figure 1) and that p66Shc is a critical component of the aging pathway. This one is triggered by the accumulation of oxidation damaged products (mainly proteins) that induces UPR and activates a specific p53 pathway that depends on the p66Shc redox activity and the p44 short isoform.

As consequence, the existence of two different and not redundant p53 pathways explains why the abrogation of the p53 aging function, as it occurs in p66Shc null mice, does not increase tumour formation.
